# Maternal and child health services during the COVID-19 pandemic in India: an interrupted time-series analysis

**DOI:** 10.3389/fgwh.2025.1578259

**Published:** 2025-11-17

**Authors:** Stuti Tripathi, Pravin Kumar Singh, Lucky Singh, Ravleen Kaur Bakshi, Tulsi Adhikari, Saritha Nair, Kh. Jitenkumar Singh, Ashoo Grover, Saurabh Sharma

**Affiliations:** 1 ICMR-National Institute for Research in Digital Health and Data Science, New Delhi, India; 2 ICMR-Headquarters, New Delhi, India.

**Keywords:** coronavirus disease, health management information system, India, maternal and child health, interrupted time series analysis

## Abstract

**Background:**

The COVID-19 pandemic posed significant challenges to healthcare systems worldwide. Maintaining essential health services, including maternal and child health (MCH), while addressing the pandemic is an enormous task. This study aimed to assess the impact of the COVID-19 pandemic on the utilization of MCH services in India's public primary care. It extends prior work by applying nationwide HMIS data within an interrupted time-series framework with seasonal and ARMA adjustments to estimate counterfactual trends, thereby providing national-level insights into both immediate and evolving disruptions.

**Methods:**

A retrospective analysis using Health Management Information System (HMIS) data examined 12 indicators of service utilization, covering maternal health, child health, deliveries, and newborn care. Interrupted time-series analysis compared pre-pandemic (April 2017–March 2020) and pandemic (March 2020–May 2021) was performed using Ordinary Least Squares (OLS) and Generalized Least Squares (GLS) regression models, adjusting for seasonality and autocorrelation with ARMA terms.

**Results:**

Antenatal care (ANC) registrations decreased by 346,420 cases (−12.8%, *p* = 0.026) following the onset of the pandemic, with no significant recovery in the subsequent months. Tetanus toxoid vaccinations also declined markedly, with Td1 and Td2 falling by 276,152 (−13.9%, *p* = 0.029) and 306,607 (−16.9%, *p* = 0.010) cases, respectively, and remaining consistently below expected levels. Institutional deliveries dropped by 272,441 (−13.7%, *p* = 0.067), while home deliveries attended by skilled birth attendants decreased by 5,054 cases (−22.8%, *p* = 0.014). Child health services, including referrals to Special Newborn Care Units (SNCUs) and inborn admissions, were also lower than anticipated (−20.4% and −19.2%, respectively), though these changes were not statistically significant. Among all indicators, the largest and most persistent disruptions occurred in obstetric complications (maximum decline during Winter 2020–21) and SNCU inborn admissions (also at their lowest in Winter 2020–21). These two services showed minimal signs of recovery throughout the study period, underscoring the particular vulnerability of emergency obstetric and neonatal care during public health crises.

**Conclusions:**

The COVID-19 pandemic caused declines in MCH service utilization, with varying recovery across indicators. While services like antenatal care and vaccinations showed some stabilization over time, child health admissions and obstetric complications remained below pre-pandemic trends. Strengthening healthcare systems to maintain essential services and support recovery during and after public health emergencies is critical.

## 1 Introduction

Coronavirus disease 2019 (COVID-19), caused by severe acute respiratory syndrome coronavirus 2 (SARS-CoV-2) (1), was a highly contagious and impactful viral pneumonia that emerged as a global health crisis since its first appearance in Wuhan, China, in December 2019 (2, 3). The virus rapidly spread worldwide, creating unprecedented public health challenges across diverse healthcare systems, with particularly severe implications in low- and middle-income countries (LMICs) such as India (4).

One of the populations particularly vulnerable to the direct and indirect effects of COVID-19 was pregnant women (5), whose health was influenced by physiological, immunological, anatomical, and hormonal shifts associated with pregnancy (6). Experience from previous large-scale outbreaks, such as severe acute respiratory syndrome (SARS), Middle East respiratory syndrome (MERS), H1N1 influenza, and Ebola virus disease (EVD) (7), showed that pregnant women faced elevated risks of adverse health outcomes, including renal failure, sepsis, and maternal mortality, as well as pregnancy-specific complications such as spontaneous abortion (8), preterm birth (9), and intrauterine growth restriction (10). In India, as in other LMICs, the COVID-19 pandemic heightened these risks due to significant disruptions in antenatal and perinatal care, critical for maternal and neonatal health.

The pandemic severely affected healthcare systems' capacity to deliver maternal and perinatal services effectively (11). In India, healthcare infrastructure limitations, human resource constraints, supply chain disruptions, and redirection of medical resources towards COVID-19 care created barriers to accessing routine maternal health services (12). These barriers were further exacerbated by movement restrictions, reduced availability of public transport, and widespread fear of contracting COVID-19 at health facilities, which further reduced antenatal care (ANC) and intrapartum care uptake (13). Consequently, maternal and neonatal mortality rates in India were at risk of reversal after years of steady progress. For example, in Sant Kabir Nagar district, Uttar Pradesh, there was a 22.91% decline in antenatal care services, a 2.26% decrease in institutional deliveries, and a drop of over 20% in immunization coverage—disruptions that are known contributors to preventable maternal and neonatal deaths (14). These setbacks jeopardized the substantial strides made toward achieving Sustainable Development Goal (SDG) 3.1, which aims to reduce maternal mortality to fewer than 70 deaths per 100,000 live births and neonatal mortality to fewer than 12 deaths per 1,000 live births by 2030 (15).

Despite extensive research on the impact of COVID-19 on maternal healthcare globally, there remained limited empirical evidence on how the pandemic affected maternal health service utilization and perinatal outcomes in India. While interrupted time-series (ITS) analyses have been conducted in other contexts to examine service disruptions, none have used India's nationwide Health Management Information System (HMIS) data, focusing specifically on maternal and child health (MCH) services. This study applied an ITS approach to monthly HMIS indicators with seasonal and ARMA adjustments to estimate counterfactual trends, thereby capturing both immediate and evolving disruptions. In doing so, it contributes unique evidence by leveraging national-level routine health information, complementing localized studies, and providing policymakers with a comprehensive assessment to strengthen maternal and neonatal health services during future public health emergencies.

## 2 Methods

### 2.1 Study design

This study employed a comparative interrupted time-series (ITS) design over a 50-month period, covering the pre-pandemic (April 2017–March 2020) and pandemic (March 2020–May 2021) phases. We retrospectively analyzed data from standardized reports of India's Health Management Information System (HMIS).

### 2.2 Data source

Data were taken from India's HMIS, a standardized platform used nationwide. The HMIS is a well-established reporting system used by all the states and union territories of India and is available through the MoHFW (Ministry of Health and Family Welfare in India). The information from HMIS is uploaded on a routine basis from the entire health unit across the nation. For this analysis, we extracted monthly indicator-level HMIS data for April 2017–May 2021 from the Reproductive and Child Health (RCH) reports under HMIS and included indicator-wise data at the national (all-India) level. Microdata for selected MCH indicators was obtained in CSV/Excel format from the HMIS website (https://hmis.mohfw.gov.in/#!/standardReports).

### 2.3 Study variables

The study focused on 12 MCH indicators obtained from HMIS data. Detailed information on these indicators is provided in Table 1.

**Table 1:** List of indicators, definitions, and abbreviations included in the analysis.

S. No.	Indicator name	Definition	Abbreviation
1.	Pregnant women (PW) registered for ANC	Number of pregnant women registered for antenatal care (ANC)	ANC Registration
2.	Number of PW given Td1	Number of pregnant women receiving the first dose of tetanus toxoid (Td) vaccine	Td1
3.	Number of PW given Td2	Number of pregnant women receiving the second dose of tetanus toxoid (Td) vaccine	Td2
4.	Number of PW given Td Booster	Number of pregnant women receiving the Td booster dose	Td Booster
5.	Number of PW given 180 Iron Folic Acid	Number of pregnant women receiving 180 tablets of Iron Folic Acid (IFA) for anemia prevention	IFA
6.	Number of cases of pregnant women with Obstetric Complications attended	Number of pregnant women attended for any obstetric complications	Obstetric Complications
7.	Number of pregnant women screened for HIV	Number of pregnant women screened for HIV during antenatal care	HIV Screening
8.	Number of Institutional Deliveries conducted, including Lower Segment Cesarean Sections (LSCS)	Number of deliveries conducted in healthcare facilities, including caesarean sections	Institutional Deliveries (Including LSCS)
9.	Number of Home Deliveries attended by Skill Birth Attendant (SBA)	Number of home deliveries attended by trained skilled birth attendants	Home Delivery SBA
10.	Number of Home Deliveries attended by non-SBA	Number of home deliveries attended by non-skilled attendants	Home Delivery Non-SBA
11.	Number of newborns admitted in Special Newborn Care Units (SNCU) referred by Accredited Social Health Activists (ASHAs)	Number of newborns admitted to SNCU referred by ASHAs	SNCU Admissions ASHA
12.	Total Special Newborn Care Unit Admissions Inborn	Total number of newborns admitted to SNCUs who were born in the same facility	SNCU Admissions Inborn

### 2.4 Statistical analysis

**Figure 1:**
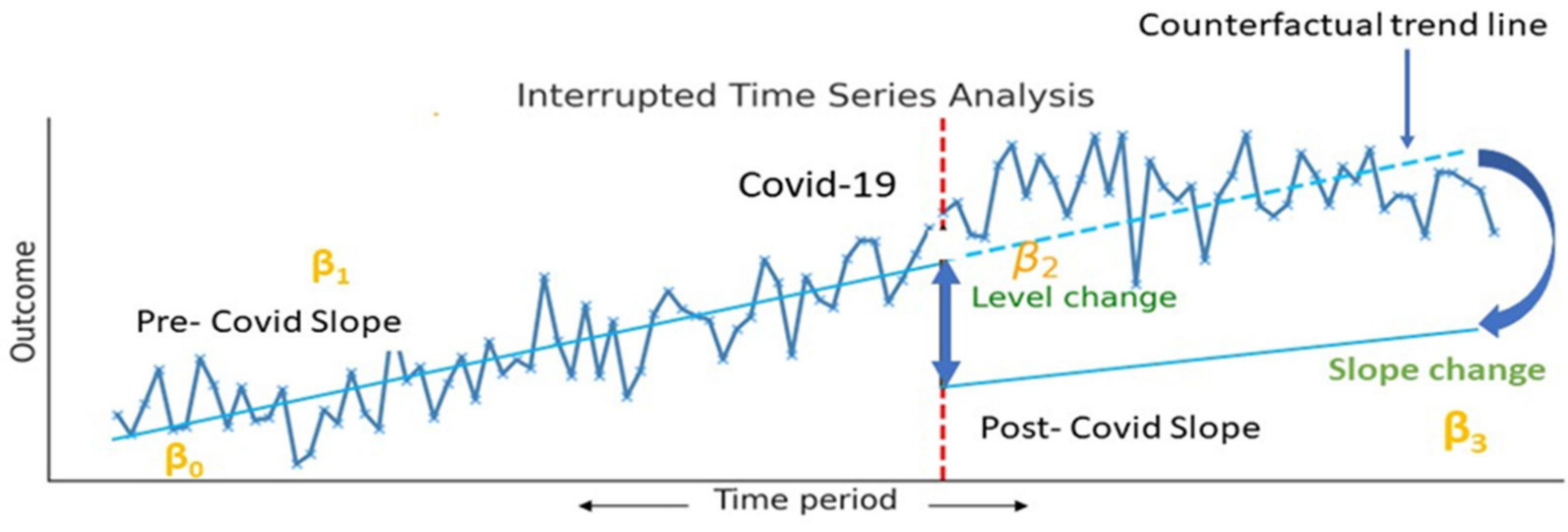
Graphical depiction of a segmented linear regression model fitted to ITS data.

Mean imputation was applied for the missing February 2020 data to maintain trend continuity. Outliers were identified using the Z-score method. Shapiro–Wilk testing verified the normality of MCH indicators pre-model fitting; non-normal indicators were log-transformed to achieve normality. Time-series data were checked for stationarity using the Augmented Dickey-Fuller test, with non-stationary indicators transformed using a moving average. Interrupted time-series analysis was conducted using ordinary least squares (OLS) and generalized least squares (GLS) models. ITS is ideal for examining time-series data across interventions, allowing for the detection of immediate and gradual changes in MCH rates due to COVID-19. Both OLS and GLS models were used for robustness against autocorrelation and moving averages (Figure 1).

The segmented regression model used was:

- **Y_t_** **=** ***β*_0_** **+** ***β*_1_ (time_t_)** **+** ***β*_2_ (level_t_)** **+** ***β*_3_(trend_t_)** **+** **ɛ_t_**

where:

- Y_t_: Outcome variable (MCH indicator) at time t,
- *β*_0_: Baseline level (Intercept) of the MCH indicator,
- *β*_1_: Pre-COVID-19 trend (Slope),
- *β*_2_: Immediate post-COVID-19 level change (Intercept Shift),
- *β*_3_: Post-COVID-19 trend change (Slope difference),
- ɛ_t_: Error term.

Model performance was evaluated using the Akaike Information Criterion (AIC) and Bayesian Information Criterion (BIC), with ARMA models (auto Arima function in R) applied for autocorrelation and seasonal adjustments.

We used a 5% significance level to analyze changes in MCH indicators between the pre-pandemic and pandemic periods, accounting for immediate, quarterly, and seasonal variations.

## 3 Results

The analysis of maternal and child health (MCH) services during the COVID-19 pandemic reveals substantial disruptions in service utilization across all major indicators, with notable seasonal variations. These findings are derived from interrupted time-series modelling that compared observed values during the pandemic to counterfactual estimates based on pre-pandemic trends.

The most severely affected MCH services during the pandemic were HIV Screening and IFA. Between March to May 2020 (Spring), HIV screening declined by 740,469 (–36.8%), and IFA coverage reduced by 382,167 (–16.7%). Although gradual recovery was observed, by December 2020 to February 2021 (Winter), HIV screening still showed a deficit of 675,812 (–30.8%) and IFA coverage remained lower by 345,103 (–14.3%) (Table 2).

**Table 2:** Interpreted time series modeling showing seasonal variation in MCH indicators during the COVID-19 pandemic, India.

Variable	Period	Observed value	Counterfactual	Absolute change	Relative change (%)
ANC Registration	March 2020 to May 2020 (Spring)	21,23,794	24,35,382	−311588	−12.8
	Jun 2020 to August 2020 (Summer)	21,78,193	24,37,533	−259,341	−10.6
	September 2020 to November 2020 (Fall)	22,32,591	24,39,685	−207,094	−8.5
	December 2020 to February 2021 (Winter)	22,86,990	24,41,836	−154,846	−6.3
Td1	March 2020 to May 2020 (Spring)	15,00,606	17,43,097	−242,490	−13.9
	Jun 2020 to August 2020 (Summer)	15,51,323	17,43,321	−191,998	−11.0
	September 2020 to November 2020 (Fall)	16,02,039	17,43,545	−141,506	−8.1
	December 2020 to February 2021 (Winter)	16,52,756	17,43,769	−91,013	−5.2
Td2	March 2020 to May 2020 (Spring)	13,07,293	15,72,663	−265,371	−16.9
	Jun 2020 to August 2020 (Summer)	13,70,449	15,73,964	−203,516	−12.9
	September 2020 to November 2020 (Fall)	14,33,605	15,75,265	−141,660	−8.9
	December 2020 to February 2021 (Winter)	14,96,761	15,76,567	−79,805	−5.1
Td Booster	March 2020 to May 2020 (Spring)	474,079	525,876	−51,797	−9.9
	Jun 2020 to August 2020 (Summer)	488,110	530,215	−42,105	−7.9
	September 2020 to November 2020 (Fall)	502,142	534,555	−32,413	−6.1
	December 2020 to February 2021 (Winter)	516,173	538,894	−22,721	−4.2
IFA	March 2020 to May 2020 (Spring)	19,05,913	22,88,080	−382,167	−16.7
	Jun 2020 to August 2020 (Summer)	19,58,871	23,28,683	−369,812	−15.9
	September 2020 to November 2020 (Fall)	20,11,829	23,69,286	−357,457	−15.1
	December 2020 to February 2021 (Winter)	20,64,787	24,09,889	−345,103	−14.3
HIV Screening	March 2020 to May 2020 (Spring)	12,72,792	20,13,262	−740,469	−36.8
	Jun 2020 to August 2020 (Summer)	13,55,885	20,74,802	−718,917	−34.7
	September 2020 to November 2020 (Fall)	14,38,977	21,36,342	−697,365	−32.6
	December 2020 to February 2021 (Winter)	15,22,070	21,97,882	−675,812	−30.8
Home Delivery Non-SBA	March 2020 to May 2020 (Spring)	57,273	82,664	−25,391	−30.7
	Jun 2020 to August 2020 (Summer)	62,110	80,670	−18,560	−23.0
	September 2020 to November 2020 (Fall)	66,946	78,675	−11,729	−14.9
	December 2020 to February 2021 (Winter)	71,782	76,681	−4,899	−6.4
Home Delivery SBA	March 2020 to May 2020 (Spring)	13,477	17,457	−3,981	−22.8
	Jun 2020 to August 2020 (Summer)	14,518	16,889	−2,371	−14.0
	September 2020 to November 2020 (Fall)	15,560	16,321	−761	−4.7
	December 2020 to February 2021 (Winter)	16,602	14,141	2,460	17.4
Institutional Deliveries (Including LSCS)	March 2020 to May 2020 (Spring)	15,15,593	17,56,057	−240,465	−13.7
	Jun 2020 to August 2020 (Summer)	15,43,510	17,73,407	−229,896	−12.9
	September 2020 to November 2020 (Fall)	15,71,428	17,90,756	−219,328	−12.3
	December 2020 to February 2021 (Winter)	15,99,346	18,08,105	−208,760	−11.6
Obstetric Complications	March 2020 to May 2020 (Spring)	130,342	167,680	−37,338	−22.3
	Jun 2020 to August 2020 (Summer)	129,273	170,585	−41,312	−24.2
	September 2020 to November 2020 (Fall)	128,204	173,489	−45,285	−26.1
	December 2020 to February 2021 (Winter)	127,135	176394	−49,259	−27.9
SNCU Admissions ASHA	March 2020 to May 2020 (Spring)	5,128	6,440	−1,312	−20.4
	Jun 2020 to August 2020 (Summer)	5,183	6,653	−1,470	−22.1
	September 2020 to November 2020 (Fall)	5,237	6,866	−1,629	−23.7
	December 2020 to February 2021 (Winter)	5,291	7,079	−1,787	−25.3
SNCU Admissions Inborn	March 2020 to May 2020 (Spring)	56,880	70,426	−13,546	−19.2
	Jun 2020 to August 2020 (Summer)	54,876	71,467	−16,591	−23.2
	September 2020 to November 2020 (Fall)	52,872	72,509	−19,637	−27.1
	December 2020 to February 2021 (Winter)	50,868	73,550	−22,683	−30.8

ANC Registration decreased by 311,588 (–12.8%) during March to May 2020 (Spring), improving gradually but still showing a deficit of 154,846 (–6.3%) by December 2020 to February 2021 (Winter).

Tetanus toxoid (Td) vaccinations were also impacted. Td1 dropped by 242,490 (–13.9%) during March to May 2020 (Spring), with the deficit narrowing to 91,013 (–5.2%) by December 2020 to February 2021 (Winter). Td2 declined by 265,371 (–16.9%) during March to May 2020 (Spring), recovering slightly to a deficit of 79,805 (–5.1%) by December 2020 to February 2021 (Winter). Similarly, Td Booster doses fell by 51,797 (–9.9%) during March to May 2020 (Spring), improving to a smaller decline of 22,721 (–4.2%) by December 2020 to February 2021 (Winter).

Institutional deliveries (including LSCS) decreased by 240,465 (–13.7%) between March to May 2020 (Spring) and remained lower by 208,760 (–11.6%) during December 2020 to February 2021 (Winter). Home Delivery SBA initially declined by 3,981 (–22.8%) during March to May 2020 (Spring) but showed a positive increase of 2,460 (17.4%) by December 2020 to February 2021 (Winter). Meanwhile, Home Delivery Non-SBA declined by 25,391 (–30.7%) during March to May 2020 (Spring), with a much smaller deficit of 4,899 (–6.4%) during December 2020 to February 2021 (Winter).

Newborn care services were notably disrupted, and these kept on declining along with obstetric complications. SNCU Admissions ASHA declined by 1,312 (–20.4%) during March to May 2020 (Spring) and further decreased to 1,787 (–25.3%) by December 2020 to February 2021 (Winter). Similarly, SNCU Admissions Inborn fell by 13,546 (–19.2%) during March to May 2020 (Spring), with the deficit widening to 22,683 (–30.8%) by December 2020 to February 2021 (Winter). Obstetric complications managed at health facilities showed a sustained decrease, with 37,338 (–22.3%) fewer cases during March to May 2020 (Spring), worsening to a deficit of 49,259 (–27.9%) by December 2020 to February 2021 (Winter).

Although the primary seasonal analysis concludes in February 2021, data from March to May 2021, the period coinciding with India's second COVID-19 wave, show a further decline in MCH service utilization across several indicators. This trend underscores the persistent vulnerability of the health system in maintaining uninterrupted services during subsequent waves of the pandemic.

**Figure 2:**
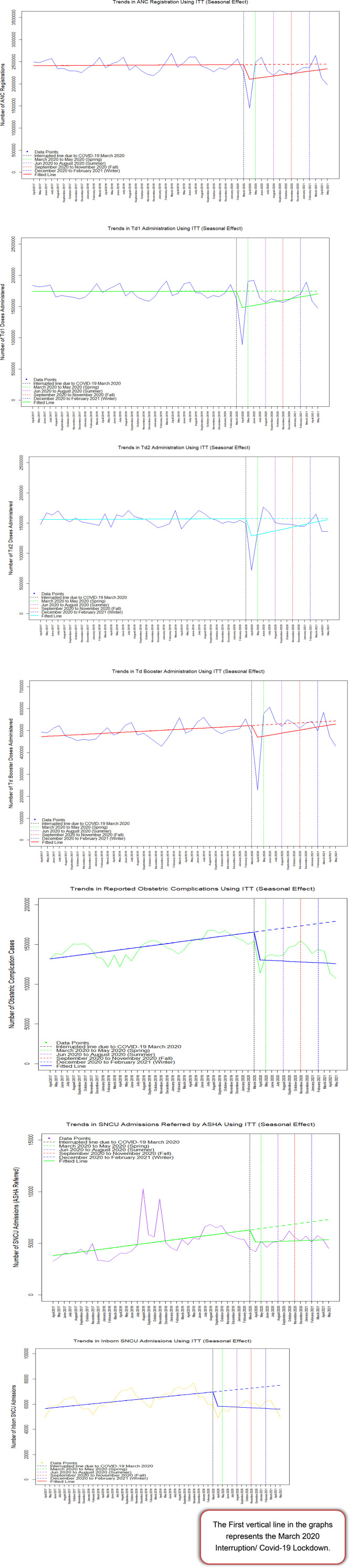
Graphical representation of interpreted time series modeling showing seasonal variation in MCH indicators during the COVID-19 pandemic, India.

**Figure A1:**
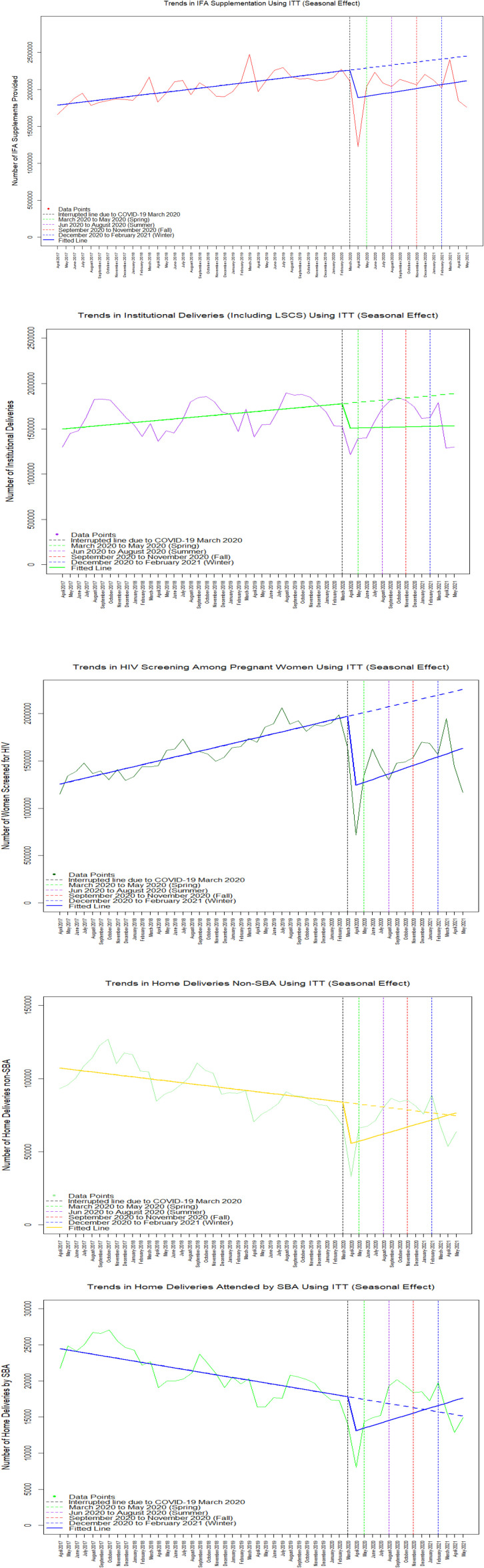
Seasonal variation and interrupted time series analysis of IFA supplementation, institutional deliveries, HIV screening, and non-SBA home deliveries in India (April 2017–March 2021).

The sharpest declines occurred during the initial lockdown period, coinciding with widespread mobility restrictions and the reallocation of healthcare resources toward COVID-19 care. Interrupted time-series seasonal graphs revealed substantial reductions in MCH service utilization during spring 2020, with variable recovery patterns in subsequent seasons. While services such as ANC registration and tetanus vaccinations gradually stabilized by winter 2020, child health indicators and obstetric complications remained well below counterfactual trends throughout the study period (Figure 2). For other variables, see Appendix Figure A1 for the full seasonal trends.

These results underscore the pandemic's substantial negative impact on MCH services and highlight the urgent need for interventions to bolster the resilience of healthcare systems during public health emergencies.

The analysis confirmed an immediate decline of 346,420 ANC Registration following the pandemic onset (*p* = 0.026), with no statistically significant trend recovery. For tetanus vaccinations, the results indicated reductions of 276,152 (*p* = 0.029) and 306,607 (*p* = 0.010) for Td1 and Td2, respectively, highlighting the sustained reductions in service delivery (Table 3).

**Table 3:** Interrupted time series analysis of maternal and child health indicators using the generalized least squares method (ARMA).

Variable	GLS Result	Value	Standard error	t value	*p*-value	L Limit	U Limit
ANC Registration	Intercept (*β*_0_)	24,08,133	83,963	28.681	0.000	22,43,569	25,72,697
	Time (β_1_)	717	3,929	0.182	0.856	−6,984	8,418
	Level (β_2_)	−346,420	149,995	−2.310	0.026	−640,405	−52,434
	Trend (β_3_)	17,416	16,214	1.074	0.288	−14,363	49,195
Td1	Intercept (β_0_)	17,40,257	67,483	25.788	0.000	16,07,993	18,72,522
	Time (β_1_)	75	3,163	0.024	0.981	−6,124	6,274
	Level (β_2_)	−276,152	122,206	−2.260	0.029	−515,671	−36,633
	Trend (β_3_)	16,831	13,106	1.284	0.206	−8,857	42,518
Td2	Intercept (β_0_)	15,56,182	64,148	24.259	0.000	14,30,454	16,81,910
	Time (β_1_)	434	3,002	0.144	0.886	−5,451	6,318
	Level (β_2_)	−306,607	114,757	−2.672	0.010	−531,527	−81,687
	Trend (β_3_)	20,618	12,395	1.664	0.103	−3,674	44,911
Td Booster	Intercept (β_0_)	470,914	24,129	19.517	0.000	423,622	518,205
	Time (β_1_)	1,446	1,131	1.279	0.207	−770	3,663
	Level (β_2_)	−58,259	43,722	−1.332	0.189	−143,953	27,436
	Trend (β_3_)	3,231	4,687	0.689	0.494	−5,956	12,418
IFA	Intercept (β_0_)	17,73,772	72,153	24.584	0.000	16,32,355	19,15,188
	Time (β_1_)	13,534	3,387	3.996	0.000	6,895	20,173
	Level (β_2_)	−390,403	132,702	−2.942	0.005	−650,495	−130,311
	Trend (β_3_)	4,118	14,114	0.292	0.772	−23,545	31,782
HIV Screening	Intercept (β_0_)	12,34,430	74,420	16.587	0.000	10,88,569	13,80,291
	Time (β_1_)	20,413	3,471	5.882	0.000	13,611	27,215
	Level (β_2_)	−758,214	128,879	−5.883	0.000	−10,10,811	−505,616
	Trend (β_3_)	9,679	14,207	0.681	0.499	−18,166	37,525
Home Delivery Non-SBA	Intercept (β_0_)	107,926	8,869	12.169	0.000	90,544	125,309
	Time (β_1_)	−665	399	−1.668	0.102	−1,446	116
	Level (β_2_)	−29,945	9,637	−3.107	0.003	−48,834	−11,056
	Trend (β_3_)	2,277	1,408	1.617	0.113	−482	5,036
Home Delivery SBA	Intercept (β_0_)	24,652	1,674	14.726	0.000	21,371	27,933
	Time (β_1_)	−189	76	−2.503	0.016	−338	−41
	Level (β_2_)	−5,054	1,967	−2.569	0.014	−8,910	−1,198
	Trend (β_3_)	537	272	1.970	0.055	3	1,071
Institutional Deliveries (Including LSCS)	Intercept (β_0_)	14,90,012	132,463	11.249	0.000	12,30,390	17,49,634
	Time (β_1_)	8,007	5,957	1.344	0.186	−3,668	19,683
	Level (β_2_)	−2,72,441	1,45,244	−1.876	0.067	−5,57,114	12,231
	Trend (β_3_)	−6,096	21,085	−0.289	0.774	−47,422	35,229
Obstetric Complications	Intercept (β_0_)	1,30,885	8,875	14.748	0.000	1,13,491	1,48,279
	Time (β_1_)	968	396	2.443	0.019	192	1,745
	Level (β_2_)	−34,689	8,808	−3.938	0.000	−51,952	−17,426
	Trend (β_3_)	−1,325	1,369	−0.967	0.339	−4,009	1,359
SNCU Admissions ASHA	Intercept (β_0_)	3,744	524	7.149	0.000	2,718	4,771
	Time (β_1_)	71	25	2.894	0.006	23	119
	Level (β_2_)	−1,206	935	−1.290	0.203	−3,039	626
	Trend (β_3_)	−53	101	−0.525	0.602	−250	144
SNCU Admissions Inborn	Intercept (β_0_)	57,233	3,344	17.114	0.000	50,678	63,787
	Time (β_1_)	347	158	2.204	0.033	38	656
	Level (β_2_)	−11,515	6,378	−1.806	0.078	−24,016	985
	Trend (β_3_)	−1,015	668	−1.520	0.135	−2,325	294

Institutional Deliveries (Including LSCS) experienced a level reduction of 272,441, which approached significance (*p* = 0.067). Similarly, Home Delivery SBA showed a significant level reduction of 5,054 cases (*p* = 0.014), while Home Delivery Non-SBA and SNCU admissions exhibited level declines, though these were not statistically significant. Obstetric complications showed the most pronounced decrease, with a level reduction of 34,689 cases (*p* < 0.001). None of the recoveries happening in the trend were significant.

## 4 Discussion

This study examined the impact of the COVID-19 pandemic on MCH services in India using HMIS data, with a focus on seasonal variations. The findings demonstrate substantial disruptions in service utilization during both the first wave (March–May 2020) (Spring) and the second wave (April–May 2021) (Spring) of COVID-19, with varying patterns of recovery across indicators. The findings reveal that both institutional and outreach MCH services were significantly affected during both pandemic waves. While most maternal and child health indicators experienced disruption during the pandemic, the most notable and hardest to recover were obstetric complications and Special Newborn Care Unit (SNCU) inborn admissions. Both indicators reached their lowest levels during Winter 2020–21 and showed minimal improvement in the months that followed. Seasonal variation in obstetric and neonatal outcomes has been reported both globally (16, 17) and in India (18, 19). Extreme temperatures, both hot and cold, have been linked to higher risks of adverse outcomes such as preterm birth, low birthweight, and stillbirth. A recent multi-site South Asian cohort (including India) demonstrated that higher second-trimester ambient temperatures increased the risk of preterm birth and low birthweight (16). Similarly, Indian studies have documented seasonal clustering of eclampsia during the monsoon and higher neonatal mortality in colder months, often driven by hypothermia, respiratory infections, and limited care-seeking (20). Such evidence supports our finding of a winter decline in obstetric complications and SNCU inborn admissions, which may partly reflect reduced conception and delivery volumes in winter, compounded by infection risks, hypothermia, and reduced health-seeking due to cold weather and accessibility barriers. This sustained decline suggests that emergency obstetric and neonatal care services were particularly vulnerable to systemic strain, mobility restrictions, and resource diversion. These findings underscore the need for targeted investments in strengthening obstetric and neonatal critical care capacity to safeguard service continuity during future public health crises.

During the first wave in 2020, disruptions were primarily attributed to the national lockdown, stringent travel restrictions, and reduced accessibility to healthcare facilities (21, 22). In contrast, the second wave (April–May 2021) saw service disruptions driven largely by the overwhelming surge in COVID-19 cases and the severity of the Delta variant, which strained institutional capacities and outreach activities alike (23, 24).

Our study revealed declines in key MCH service indicators during the pandemic, aligning with global findings. For instance, antenatal care (ANC) registrations in India missed by 13% during the spring lockdown of 2020, similar to reductions reported in South Africa, where child healthcare visits fell by over 50% during the national lockdown (12, 25). Likewise, disruptions during the Ebola outbreak in West Africa demonstrated similar service interruptions, with significant declines in antenatal and postnatal care utilization contributing to adverse maternal and child outcomes (26, 27). These parallels emphasize the vulnerability of MCH services to health crises and the critical need for system resilience.

The Government of India implemented several initiatives and policies for essential MCH services. The Janani Shishu Suraksha Karyakaram (JSSK) (28) and Pradhan Mantri Surakshit Matritva Abhiyan (PMSMA) (29) were leveraged to ensure free and equitable access to maternal healthcare (30). To address supply-side challenges, the government mobilized resources through programs such as Ayushman Bharat-Health and Wellness Centres (AB-HWCs)**,** which decentralized service delivery and facilitated continued access to primary healthcare (31). Additionally, telemedicine platforms like eSanjeevani were scaled up, enabling remote consultations and reducing the burden on physical healthcare facilities (32). However, during the COVID-19 pandemic, the delivery of these pre-existing programs was significantly disrupted, as evidenced by declines in institutional deliveries, antenatal check-ups under PMSMA, and immunization coverage, underscoring the fragility of MCH services during health crises (30).

Community-level interventions by ASHAs played a vital role in mitigating demand-side barriers. ASHAs facilitated doorstep delivery of essential supplies, created awareness about continued MCH services, and ensured the safe transport of pregnant women to healthcare facilities, even during mobility restrictions (33). These efforts resulted in partial recovery of some services, such as tetanus vaccinations and institutional deliveries, by the winter of 2020, as observed in our study.

However, significant service delivery gaps remained even after initial recovery, pointing to critical weaknesses in the system that persisted throughout the pandemic period. For example, child health services showed sustained declines of over 70% by winter, underscoring the pandemic's long-term impact. Similarly, the distribution of iron and folic acid tablets and HIV screening for pregnant women experienced significant disruptions, reflecting broader systemic challenges in preventive health services. These findings highlight areas where further intervention is needed to strengthen health service delivery.

Newborn care services were notably disrupted during the pandemic, with Special Newborn Care Unit (SNCU) admissions, both referred by ASHAs and inborn, showing consistent and progressive declines across all seasons. This sustained reduction in admissions, even after initial recovery periods, reflects the lingering impact of the pandemic on neonatal care. Similarly, the number of obstetric complications managed at health facilities also declined steadily over time, suggesting that critical maternal and neonatal health issues were not adequately addressed. The parallel trends in these two indicators point to systemic gaps in emergency obstetric and newborn care that persisted throughout the study period, warranting urgent attention to ensure these essential services are safeguarded during future health crises.

Despite these challenges, India's ability to adapt MCH services during the pandemic highlights the potential to build a resilient healthcare system through innovation and improvisation. Initiatives such as the launch of the National Telemedicine Service were instrumental in mitigating the pandemic's adverse effects (34). To ensure the seamless provision of healthcare services, the government of India has formulated advisories and guidelines, empowering state governments to develop their action plans. These guidelines have played a pivotal role in facilitating the maintenance of a continuum of care and have provided valuable support to state governments in strategizing their healthcare initiatives (35). These collective efforts align with global recommendations (36–38), including those from the World Health Organization for maintaining essential health services during public health emergencies. However, our findings, along with other studies, suggest that these commendable measures were insufficient to fully prevent service disruptions, particularly in routine immunization and maternal care. This highlights a critical implementation gap and underscores the need for more resilient and adaptable health systems at the last mile.

The disruption in MCH services observed in this study reflects a combination of supply- and demand-side challenges. On the supply side, workforce reallocation to COVID-19 care, resource shortages, and disruptions in the supply chain hindered service delivery. On the demand side, fear of infection, mobility restrictions, economic constraints, and a decline in public trust in the health system reduced healthcare-seeking behavior during the pandemic period. The erosion of trust, combined with concerns about infection risks within healthcare settings, further discouraged the timely utilization of maternal and child health services. The interplay between these factors contributed to substantial declines in ANC registrations and provision of child health services. However, Community Health Workers played a critical role by visiting antenatal and postnatal mothers at their homes to deliver essential MCH services, including weight monitoring, counseling, distribution of medications, and provision of nutritional supplements. Similar interventions have been recommended in other studies, emphasizing the importance of introducing home-based care strategies for managing high-risk pregnancies (36, 39), creating social media groups connecting healthcare providers and pregnant women to facilitate continuous support and guidance (37), and promoting teleconsultation services to maintain access to maternal and child health care during disruptions (38), as evidenced by the gradual recovery trends observed in immunization indicators among pregnant women and in institutional deliveries.

### 4.1 Limitations and strengths

This study is not without limitations. As with all secondary data analyses, our findings depend on the quality and completeness of the data extracted from the HMIS, which may vary across facilities and regions. Although HMIS captures data from both public and private healthcare facilities in India, the reporting from private facilities may not be as comprehensive or consistent as that from public facilities, potentially introducing bias or underrepresentation in our analysis of service utilization trends. Moreover, disentangling the supply- and demand-side effects of the pandemic proved challenging, and geographic differences in healthcare delivery were not analyzed. Additionally, due to data availability only through May 2021, a distinct post-pandemic recovery phase could not be evaluated.

One of the main strengths of this study is its study design. The study employed interrupted time-series (ITS) analysis, a robust quasi-experimental method, to assess the real-time effects of the COVID-19 pandemic. ITS is particularly suited to evaluating interventions or disruptions, allowing the study to capture the immediate and long-term impacts of the pandemic, such as the effects of lockdown measures and seasonal variations. This approach enables the study to confidently attribute the observed disruptions in service delivery and utilization, as well as increases in mortality rates, to the COVID-19 pandemic. These findings underscore the importance of resilient health systems capable of mitigating such disruptions during future public health emergencies. The nationwide scope of the HMIS data enhances the generalizability of our findings, providing a comprehensive view of maternal and child health service utilization across India.

## 5 Conclusion

The COVID-19 pandemic profoundly impacted maternal and child health (MCH) services in India, particularly during the initial lockdown period. Our findings reveal notable declines in key health service indicators, including antenatal care registrations, tetanus vaccinations, and child health interventions. While some services demonstrated partial recovery post-lockdown, others continued to face substantial challenges, reflecting the heterogeneous impact of the pandemic on different healthcare delivery services. Substantial challenges remain in restoring maternal and child health services to pre-pandemic levels. These disruptions were driven by both supply-side factors, such as resource reallocation to COVID-19 care and workforce shortages, and demand-side barriers, including mobility restrictions and fear of infection. The consequences of these disruptions are evident, underscoring the critical need for resilient healthcare systems that can sustain essential services during public health emergencies.

In our multivariate GLS analysis, several indicators continued to show statistically significant declines even after adjusting for seasonality and autocorrelation. Obstetric complications (*p* < 0.001), HIV screening (*p* < 0.001), and tetanus toxoid vaccinations (Td1: *p* = 0.029; Td2: *p* = 0.010) remained persistently below expected levels throughout the study period. These results highlight that, while some services, such as antenatal care registrations, showed partial recovery, critical preventive and emergency care indicators were unable to rebound, suggesting the need for targeted, sustained interventions. Moving forward, targeted interventions are required to address the lingering deficits in MCH services, with particular attention to the most severely impacted areas, such as child health and maternal immunizations. Strengthening healthcare systems to withstand future crises necessitates a dual focus: maintaining routine service delivery while responding effectively to emergencies. These lessons should inform policy frameworks to ensure that essential healthcare services remain accessible and equitable during future health crises, safeguarding progress towards maternal and child health targets.

## Data Availability

Publicly available datasets were analyzed in this study. This data can be found here: https://nrhm-mis.nic.in/hmisreports/frmstandard_reports.aspx.

## References

[1] Chen Y Li Z Zhang YY Zhao WH Yu ZY. Maternal health care management during the outbreak of coronavirus disease 2019. J Med Virol. (2020) 92:731–9. 10.1002/jmv.25787 32219871

[2] WHO. WHO Announces COVID-19 Pandemic. Geneva, Switzerland: WHO (2020).

[3] Rasmussen SA Smulian JC Lednicky JA Wen TS Jamieson DJ. Coronavirus disease 2019 (COVID-19) and pregnancy: what obstetricians need to know. Am J Obstet Gynecol. (2020) 222:415–26. 10.1016/j.ajog.2020.02.017 32105680 PMC7093856

[4] Kotlar B Gerson EM Petrillo S Langer A Tiemeier H. The impact of the COVID-19 pandemic on maternal and perinatal health: a scoping review. BMC Reprod Health. (2021) 18:10. 10.1186/s12978-021-01070-6 PMC7812564 33461593

[5] Papageorghiou AT Deruelle P Gunier RB Rauch S García-May PK Mhatre M Preeclampsia and COVID-19: results from the INTERCOVID prospective longitudinal study. Am J Obstet Gynecol. (2021) 225:289.e1–289.e17. 10.1016/j.ajog.2021.05.014 34187688 PMC8233533

[6] Zhao X Jiang Y Zhao Y Xi H Liu C Qu F Analysis of the susceptibility to COVID-19 in pregnancy and recommendations on potential drug screening. Eur J Clin Microbiol Infect Dis. (2020) 39:1209–20. 10.1007/s10096-020-03897-6 32328850 PMC7178925

[7] Mehand MS Al-Shorbaji F Millett P Murgue B. The WHO R&D blueprint: 2018 review of emerging infectious diseases requiring urgent research and development efforts. Antivir Res. (2018) 159:63–7. 30261226 10.1016/j.antiviral.2018.09.009 PMC7113760

[8] Payne DC Iblan I Alqasrawi S Al Nsour M Rha B Tohme RA Stillbirth during infection with middle east respiratory syndrome coronavirus. J Infect Dis. (2014) 209:1870–2. 24474813 10.1093/infdis/jiu068 PMC4618552

[9] Olgun NS. Viral infections in pregnancy: a focus on ebola virus. Curr Pharm Des. (2018) 24:993–8. 29384053 10.2174/1381612824666180130121946 PMC6419752

[10] Wong SF Chow KM Leung TN Ng WF Ng TK Shek CC Pregnancy and perinatal outcomes of women with severe acute respiratory syndrome. Am J Obstet Gynecol. (2004) 191:292–7. 15295381 10.1016/j.ajog.2003.11.019 PMC7137614

[11] Nakate MG Mackay S Ndirangu-Mugo E Fleming V. Experiences of mothers and significant others in accessing comprehensive healthcare in the first 1000 days of life post-conception during COVID-19 in rural Uganda. BMC Pregnancy Childbirth. (2022) 22:938. 36522709 10.1186/s12884-022-05212-x PMC9754309

[12] Aranda Z Binde T Tashman K Tadikonda A Mawindo B Maweu D Disruptions in maternal health service use during the COVID-19 pandemic in 2020: experiences from 37 health facilities in low-income and middle-income countries. BMJ Glob Heal. (2022) 7:e007247. 10.1136/bmjgh-2021-007247 PMC8753094 35012970

[13] Bankar S Ghosh D. Accessing antenatal care (ANC) services during the COVID-19 first wave: insights into decision-making in rural India. BMC Reprod Health. (2022) 19:158. 10.1186/s12978-022-01446-2 PMC9264734 35804394

[14] Singh AK Jain PK Singh NP Kumar S Bajpai PK Singh S Impact of COVID-19 pandemic on maternal and child health services in Uttar Pradesh, India. J Family Med Prim Care. (2021) 10(1):509–13. 10.4103/jfmpc.jfmpc_1550_20 34017779 PMC8132817

[15] Chmielewska B Barratt I Townsend R Kalafat E van der Meulen J Gurol-Urganci I Effects of the COVID-19 pandemic on maternal and perinatal outcomes: a systematic review and meta-analysis. Lancet Glob Health. (2021) 9:e759–72. 33811827 10.1016/S2214-109X(21)00079-6 PMC8012052

[16] Shankar K Hwang K Westcott JL Saleem S Ali SA Jessani S Associations between ambient temperature and pregnancy outcomes from three South Asian sites of the global network maternal newborn health registry: a retrospective cohort study. BJOG: Int J Obstet Gynaecol. (2023) 130(Suppl 3):124–33. 10.1111/1471-0528.17616 PMC10843605 37581948

[17] Lee SJ Steer PJ Filippi V. Seasonal patterns and preterm birth: a systematic review of the literature and an analysis in a London-based cohort. BJOG: Int J Obstet Gynaecol. (2006) 113(11):1280–8. 10.1111/j.1471-0528.2006.01055.x 17120349

[18] Gupta A. Seasonal variation in infant mortality in India. Popul Stud (Camb). (2022) 76(3):535–52. 10.1080/00324728.2022.2112746 36106801

[19] Subramaniam V. Seasonal variation in the incidence of preeclampsia and eclampsia in tropical climatic conditions. BMC Women’s Health. (2007) 7:18. 10.1186/1472-6874-7-18 17937797 PMC2169212

[20] Bang AT Reddy HM Baitule SB Deshmukh MD Bang RA. The incidence of morbidities in a cohort of neonates in rural Gadchiroli, India: seasonal and temporal variation and a hypothesis about prevention. J Perinatol. (2005) 25(Suppl 1):S18–28. 10.1038/sj.jp.7211271 15791274

[21] Kc A Gurung R Kinney MV Sunny AK Basnet O Paudel P Effect of the COVID-19 pandemic response on intrapartum care, stillbirth, and neonatal mortality outcomes in Nepal: a prospective observational study. Lancet Glob Health. (2020) 8:e1273–81. 32791117 10.1016/S2214-109X(20)30345-4 PMC7417164

[22] WHO; UNICEF; UNFPA; World Bank Group; UNDESA/Population Division. Trends in Maternal Mortality 2000 to 2020. Geneva, Switzerland: World Health Organization (2023).

[23] Ward ZJ Atun R King G Sequeira Dmello B Goldie SJ. Simulation-based estimates and projections of global, regional and country-level maternal mortality by cause, 1990–2050. Nat Med. (2023) 29:1253–61. 37081226 10.1038/s41591-023-02310-x PMC10202807

[24] Sharma S Aggarwal S Kulkarni R Kumar D Mishra BK Dwivedi GR Challenges in accessing and delivering maternal and child health services during the COVID-19 pandemic: a cross-sectional rapid survey from six states of India. Int J Environ Res Public Health. (2023) 20:1538. 10.3390/ijerph20021538 36674296 PMC9865999

[25] Roberton T Carter ED Chou VB Stegmuller AR Jackson BD Tam Y Early estimates of the indirect effects of the COVID-19 pandemic on maternal and child mortality in low-income and middle-income countries: a modelling study. Lancet Glob Health. (2020) 8(7):e901–8. 32405459 10.1016/S2214-109X(20)30229-1 PMC7217645

[26] Brolin Ribacke KJ van Duinen AJ Nordenstedt H Höijer J Molnes R Froseth TW The impact of the West Africa Ebola outbreak on obstetric health care in Sierra Leone. PLoS One. (2016) 11(2):e0150080. 10.1371/journal.pone.0150080 26910462 PMC4766087

[27] Nyenswah TG Kateh F Bawo L Massaquoi M Gbanyan M Fallah M Ebola and its control in Liberia, 2014–2015. Emerg Infect Dis. (2016) 22(2):169–77. 10.3201/eid2202.151456 26811980 PMC4734504

[28] Janani Shishu Suraksha Karyakaram (JSSK). (2025). Available online at: https://nhm.gov.in/index4.php https://nhm.gov.in/index4.php (Accessed January 20,2025).

[29] Pradhan Mantri Surakshit Matritva Abhiyan (PMSMA). (2025). Available online at: https://pmsma.mohfw.gov.in/about-scheme/ (Accessed January 20,2025).

[30] Jain G Prajapati RKP Bisen V. Assessing the impact of the COVID-19 pandemic on maternal and child health services: a comprehensive analysis of government initiatives in northern India. Cureus. (2024) 16(3):e56313. 10.7759/cureus.56313 38629024 PMC11020602

[31] Ayushman bharat health and wellness centres. Govt of India. Ministry of Health and Family Welfare (2019). Available online at: https://abhwc.nhp.gov.in/assets/hwcpdf/Reforms_Booklet_HWC_English_updated_14th_Sep_2021.pdf

[32] eSanjeevani—National Telemedicine Service of India. Ministry of Health and Family Welfare (MoHFW). Government of India (2024). Available online at: https://esanjeevani.mohfw.gov.in/#/ (Accessed January 20, 2025).

[33] NITI Aayog. “Mitigation and Management of COVID-19”, 2022. Available online at: https://www.niti.gov.in/sites/default/files/2023-03/Mitigation-and-Management-of-COVID-19-Compendium-of-Ayush-based-Practices.pdf (Accessed January 20, 2025).

[34] ESanjeevani. Guidelines for Telemedicine Services, Ministry of Health & Family Welfare (MoHFW). Government of India (2019), Available online at: https://esanjeevani.mohfw.gov.in/assets/guidelines/Guidelines_for_Telemedicine_Services.pdf (Accessed: January 20, 2025).

[35] Guidance Note on Provision of Reproductive, Maternal, Newborn, Child, Adolescent Health Plus Nutrition (RMNCAH+N) Services During & Post COVID-19 Pandemic. Geneva: World Health Organization. Available online at: https://www.mohfw.gov.in/pdf/UpdatedAdditionalguidelinesonrationaluseofPersonalProtectiveEquipmentsettingapproachforHealthf (Accessed May 20, 2025).

[36] Royal College of Obstetricians & Gynaecologists. Coronavirus Infection and Pregnancy; 2020.

[37] American College of Obstetricians & Gynaecologists. Coronavirus (COVID-19), Pregnancy, and Breastfeeding. Geneva: American College of Obstetricians & Gynaecologists (2020).

[38] World Health organization. Pregnancy, Childbirth, Breastfeeding and COVID-19. Geneva: World Health organization (2020).

[39] Nguyen NH Nguyen AQ Duong PX Van Nguyen T. Using emerging telehealth technology as a future model in Vietnam during the COVID-19 pandemic: practical experience from phutho general hospital. JMIR Form Res. (2021) 5:e27968. 10.2196/27968 34078590 PMC8221284

